# The Impact of Diglossia on Executive Functions and on Reading in Arabic

**DOI:** 10.3390/brainsci14100963

**Published:** 2024-09-25

**Authors:** Raphiq Ibrahim

**Affiliations:** Edmond J. Safra Brain Research Center for the Study of Learning Disabilities, University of Haifa, Haifa 3498838, Israel; raphiq@psy.haifa.ac.il

**Keywords:** diglossia, bilingualism, metacognitive abilities, executive functions, cognitive flexibility

## Abstract

Background: In contrast to most other languages, where the spoken and written words are similar, children that have mastered Spoken Arabic (SA) learn to read a new written form of Arabic usually called Literary Arabic (LA). This phenomenon is called “diglossia”. Methods: Based on a series of studies comparing monolingual Arabic speaking and bilingual children, it has been suggested that Arabic speaking individuals develop metacognitive abilities that are considered bilinguals de facto. Some of the cognitive functions that would seem to benefit from fluency in more than one language are metalinguistic and metacognitive awareness. Results: This review article summarizes the results of studies on the relationship between bilingualism, diglossia and executive functions (EFs) which involve metacognitive awareness, selective attention, control of inhibition and cognitive flexibility as well as working memory (phonemic manipulation and metalingual performances). Conclusions: The findings are in line with research results that have shown that bilingualism has a positive effect on the functioning of an individual’s attentional system across the lifespan. The neural basis of diglossia in Arabic, as well as the conclusions and implications drawn from the impact of diglossia on EF and on reading in Arabic, are discussed.

## 1. Introduction

The spoken form of Arabic, *ammia* (SA), the local dialect, is used by speakers of the language in a specific geographic area for daily verbal communication, and it is the specific dialect of the native language of virtually all Arabic speakers. Eviatar and Ibrahim (2000) claimed that “it differs from the literary form, *fusha* (LA), known as ‘Modern Standard Arabic’ (MSA), which is the language that all speakers of Arabic the world over read and write. Thus, from an ecological point of view, SA and LA can be considered an example of diglossia: a social environment in which a community uses two forms of the same language concomitantly” [1]. From a linguistic point of view, two different systems (spoken and literary) exist with differences in linguistic structure for all linguistic domains: phonology, morphology, syntax and semantics. There is a lexical status for the *ammia* (SA) and *fusha* (LA). This dictionary dimension is the most prominent aspect of the difference between these forms, which can be both auditorally and visually distinguished as follows. 1. Identical words: These are words that are identical between the colloquial and classical languages, such as: شارع: “shari”—شارع “shari” (street), ولد “walad”—ولد “walad” (boy). 2. Similar words: These are words that are similar in letters but differ in the order of letters, such as: برتقال “bortoqal”—برتقان “bortqan” (orange), حائط “haet”—حيط “het” (wall). 3. Different words: These are words that do not have any similarity in letters. In terms of difficulty, they are more difficult than identical words, such as: هاتف “hatef”—تلفون “talafon” (telephone), شرفة “shurfa”—برندة “baranda” (balcony). Hence, literate Arabic speakers could be considered de facto bilinguals [2,3,4].

Previous studies using behavioral measures have suggested that the cognitive system treats LA differently than SA. Both LA and Hebrew (L2) are formally acquired by Arabic speakers later in life [4]. Ibrahim and Aharon-Peretz (2005) wrote that “they have used auditory lexical decision for assessing semantic priming, have suggested that SA behaved as the dominant language variety relative to LA and Hebrew as attested by the magnitude of the priming effects. Inversely, studies using visual presentation of LA, SA and Hebrew words showed that LA presented as the dominant language variety, with the SA words presenting as LA low frequency words”. Based on such evidence, Ibrahim and Aharon-Peretz (2005) hypothesized that “due to the diglossic nature of Arabic, the status of SA and LA would be modality-dependent with SA functioning like an L1 and LA as L2 in the auditory modality and LA functioning as an L1 and SA as an L2 in the visual written modality” [4]. From a related view, Alrwaita, Houston-Price and Pliatsikas (2023) focused on the cognitive abilities of speakers who use different varieties of the same language, such as in bidialectalism and diglossia [5]. According to these authors, diglossia (or bidialectalism), like bilingualism, involves the concurrent use of two language varieties. However, these situations have been suggested to offer fewer or more limited opportunities for mixing or switching between the varieties, potentially leading to different cognitive outcomes than those observed in bilingualism. They showed evidence on how bidialectalism and diglossia affect cognition and evaluate these findings in light of theories regarding the cognitive impacts of bilingualism.

Along these lines, neurobiological studies (fMRI, ERP) have revealed that for diglossic native Arabic speakers, the first-acquired SA could ‘look’ like an L2 in the written modality. These results strengthen the assumption that SA and LA performance might change as a function of the language modality used [6,7,8].

The goal of this review article is to define the behavioral and neural signature for diglossia, focusing on its relation to executive functions (EFs). Also, the article tries to examine if EF in the Arab population differs from that of other populations in relation to reading (domain specific) and other cognitive skills (domain general) [4].

## 2. Background

### 2.1. Bilingualism and the Brain

Three theories have been suggested to explain language representation in the brain. These theories have focused on the linguistic knowledge which purports there is the “linguistic domain” where first language (L1) and second language (L2) representations would be sustained by the same brain area [9,10,11]). Brain activity studies have revealed that the same brain regions are responsible for the representation of multiple languages of bilinguals regardless of the grade of similarity between these languages [12]. Klein et al. (1994) reported that “according to this theory, the organization of the lexical representations of the two languages (L1 and L2) would be governed by variables such as grammatical class and semantic category regardless of language membership”. Klein, Milner, Zatorre, Zhao, and Nikelski (1999) compared the cerebral organization of two typologically distant languages: English and Mandarin Chinese [13]. They assumed that “subjects, proficient in both languages, learned their L2s during adolescence”. The rationale was that “Mandarin was chosen in that it differs from English in its specified use of pitch and tone” and “the study examined the influence of linguistic structure on cerebral blood flow (CBF) patterns in subjects when they performed a noun-verb generation task”. The task conditions consisted of repeating nouns in Mandarin, repeating nouns in English, generating a verb for a noun in Mandarin, and generating a verb for a noun in English. Overall, “the pattern of CBF increase seen in response to the L1 was strikingly similar to that seen for the L2”. This finding led to the conclusion that fluent bilinguals, who use both languages in daily life, utilize common cortical areas for their lexical search. The results from an event-related potential (ERP) and magnetic resonance imaging (fMRI) showed a shared neural mechanism for the processing of native and second languages [14]. Moreover, Illes and his colleagues (1999) examined brain activation in bilingual participants who sequentially learned English and Spanish (or vice versa) [15]. They found that “the participants became fluent in their L2s a decade after L1 acquisition but were proficient in both languages [15]. Subjects were presented with 480 concrete and abstract English nouns and their Spanish translations. Participants performed tasks that required semantic and non-semantic decisions about those words. The semantic activation for both languages occurred in the same cortical locations. Further, no activation difference was observed in a direct comparison of semantic judgments in English and Spanish”. They suggested that “according to the resolution provided by functional MRI, a common neural system mediates semantic processes for the two languages in the bilingual brain”. They concluded that “learning a new language after puberty does not require the addition of a new semantic processing system or the recruitment of new cortical regions”.

The second theory called the “language-membership principle” indicates that bilingual persons could have distinct cortical language areas in the brain; that is, L1 and L2 representations would be sustained by different brain areas, meaning that a bilingual aphasic may selectively recover one language while the other is lost [16]. Cases of selective aphasia and other results from neuro-imaging techniques were described by Dehaene and his colleagues [17]. They found a dissociation between cortical areas involved in French (L1) and English (L2) languages. The regions of the left superior temporal sulcus, superior and middle temporal gyri showed consistent activation across subjects during the presentation of L1. Most localization theories of language suggested evidence for distinct brain regions in the representations of multiple languages of a bilingual [18].

A third theory claimed that both the “language membership” and “linguistic domain” would affect the way L2 information is represented in the brain. Case studies of bilingual aphasia patients have indicated dissociation and a double dissociation between a first language (L2) and second language (L1). However, researchers must keep in mind that lesions in the brain are often widespread. Two different single case studies reported by Ibrahim (2008, 2009) described the performance of two Arab–Hebrew bilinguals who suffered a brain lesion tumor and had undergone surgery [4,19]. In both studies, the linguistic abilities were impaired as a result of brain lesions and exhibited different symptomologies in both languages. In the first case, the patient (MH) was an Arabic–Hebrew bilingual who suffered from brain lesions following a brain tumor, evincing a dissociation between his ability to perceive and produce his second language (Hebrew). In the second case, another patient (MM) who suffered brain damage following a hemorrhage evinced a complementary picture with a double dissociation to the MH case report [4,19].

### 2.2. Behavioral and Neural Basis of Diglossia in Arabic

Although efforts have been devoted to a psycholinguistic investigation of the relationship between the two Arabic varieties, few studies have addressed the question of the brain basis of diglossia.

Based on the hypothesis that the processing of the two varieties of Arabic in the brain of literate native speakers of Arabic might mimic that of two separate languages, behavioral findings, using event-related potential (ERP) [19] and fMRI [7] technique analysis, have suggested that Literary Arabic presents more similarities with Hebrew than with Spoken Arabic in written Arabic word processing in adult subjects. The statistical comparisons of the ERP signals from SA, LA and Hebrew revealed reliable significant response differences between Spoken Arabic and both Literary Arabic and Hebrew. The fact that differences between the two forms of Arabic appeared both in terms of response speed and brain response amplitude is compatible with the history of acquisition of the phonological representations and words in these different language varieties and with the frequency of exposure to them in the auditory modality in everyday life [20]. The overall findings confirm the dominance of SA in the auditory modality and support results from previous studies suggesting that SA words behave as first language ones [4,6,21]. As for the visual lexical decision task, the results showed that using the same stimuli led to faster recognition of LA words. In parallel, ERP analysis showed a modulation of the P2-N2 components which reflected the ease with which words were identified in the different language conditions [22]. Hence, Khateb and his colleagues (2016) suggested that these differences are mainly due to the visual familiarity/frequency of exposure to words in LA [22]. In the functional brain imaging (fMRI) literature, one of the explanations raised to account for the differences observed between the processing of L2 vs. L1 written words was the difference in proficiency in L2 compared to L1, the age of acquisition of L2, or the difference in subjective frequency of L2 words [23,24,25]. The pattern of RTs and electrophysiological response differences in the auditory and visual lexical decision tasks using SA, LA and Hebrew words confirmed the prediction and previous findings in the literature that the status of SA and LA in the cognitive system of native literate Arabic speakers is modality-dependent. Along these lines, a recent fMRI study that analyzed the processing of LA, SA and Hebrew words using a semantic categorization task confirmed these observations [7]. Nevat and his colleagues found that RTs were faster for Literary Arabic than for Spoken Arabic and Hebrew. The comparison of brain response between SA and LA revealed differences that mimicked the activation patterns found in comparisons of second language vs. first language word conditions. In particular, an increase in activation was found in SA relative to LA (and not the inverse) in several language and left hemisphere areas. In a recent paper, Khateb and Ibrahim (2022) proposed that literate native speakers of Arabic who master the use of both SA and LA in everyday life function with two first languages: Spoken Arabic in the auditory modality and Literary Arabic in the visual written modality [9]. Furthermore, they proposed that the cognitive status of each of the Arabic varieties seems to depend on several parameters that include (among other things) the nature of the task’s demands, the linguistic register, the individual’s proficiency in both varieties, the modality of presentation of the stimuli (auditory vs. visual) and the type of processing (reception vs. production, etc.).

## 3. Bilingualism, Diglossia and Executive Functions (EFs)

Executive function is a term describing cognitive processes that underlie learning, which include the speed of processing, inhibition, impulse control, working memory, shifting, organization, planning, emotional regulation, verbal fluency, and problem solving [26,27]. These abilities were found to be strongly related to school readiness and academic outcomes across cultural and geographical backgrounds [28]. According to Green’s Joint Activation Model [29,30], two languages are active in the brain of a bilingual, although it would be necessary to use a mechanism to inhibit the activation of the non-target language during communication. The adaptive control hypothesis (ACH) suggested later by Green and Abutalebi (2013) explores how bilingual language use and executive functions, including attentional processes, can adapt depending on different interactional contexts [30]. Green and Abutalebi (2013) hypothesized that for bilingual children, various interactional configurations are built upon a bi-/multilingual interactional framework composed of key elements from both home and school environments throughout development. The distinctive features of each context gradually contribute to the child’s cognitive language abilities and executive functions. In other words, at home, children may be exposed to varying degrees of two or more languages. Conversely, when children begin school, they are exposed to one or more languages based on the educational program. Based on their results, the authors assumed that “bi-/multilingual interactional profiles, derived from different home and school contexts, influence task performance related to various attentional processes”.

According to Bialystok (1999), bilinguals have an advantage in executive functions because they must have constant access to information in the working memory and the ability to monitor what happens during the interaction, so-called “cognitive flexibility (“cognitive flexibility” refers to the ability to switch between tasks, inhibition to the ability to suppress dominant responses and monitoring to the ability to update information in the working memory.)” [31]. Also, authors assumed that bilingualism has a positive effect on the functioning of an individual’s attentional system across the lifespan [32].

### 3.1. Bilingualism, Working Memory and Metalinguistic Performance

Some of the cognitive functions that would seem to benefit from the knowledge of several languages are metalinguistic and metacognitive awareness (the ability to represent abstract and symbolic concepts), and specifically, bilingualism was found to improve executive functioning. In that regard, Prior et al. (2016) compared bilingual Russian–Hebrew-speaking children from two age groups (preschool and sixth grade) with their monolingual Hebrew-speaking peers, which were matched for sociocultural background [33]. The bilingual children’s vocabulary knowledge in both languages was measured objectively as an index of proficiency, and children were classified as balanced or unbalanced bilinguals. Participants performed a flanker task, measuring both inhibitory ability and cognitive flexibility. The results reveled that sixth-grade bilinguals showed some advantages over monolinguals in inhibition, but these were limited to the balanced bilinguals, who were able to achieve and maintain comparable levels of proficiency in their two languages. These findings suggest that only the demands posed by balanced bilingualism and strong competition between the two languages might lead to EF advantages, specifically in inhibition. In their recent study, Gade et al. (in press) assessed participants of five bilingual populations who together spoke five different languages [34]. They tested the hypothesis related to the language switch costs and language dominance effect by measuring EF indexes for processes of transient, reactive inhibitory language control, and sustained, proactive inhibitory language control. The researchers compared correlations among language capability measures, sociodemographic variables, and working memory. They observed benefits of language fluency measures that bolster the conceptualization of bilingualism as a multifaceted experience and concluded that bilingualism led to comparable outcomes in cognitive flexibility. In regard to diglossia and its relation to cognitive functions, it is unknown whether the diglossic challenge in Arabic-speaking children increases the utilization of EF in general.

Saiegh-Haddad (2003) hypothesized that cognitive load results from phonological distance slows reading acquisition in Arabic compared to other languages [3]. The effect of phonological distance on phonological awareness has been studied, but not its contribution to specific components, including lexical distance, stimulus type (words versus non-words) and tasks involving working memory (target phoneme location in tasks and stimulus length on phonological awareness). The term phonological memory has been used to denote the idea that reading measures (decoding, reading comprehension, and reading time) are mediated by two working memory systems: the phonological loop memory component and the visuospatial sketchpad component that performs a similar function for visual information. These two subsystems provide active storage that is capable of combining information from sensory input and the central system [35]. In a related study, Ibrahim (2011) investigated whether working memory (supra-lexical factor) affects metalingual performance, which is critical for the development of reading skill in Arabic language readers, and whether this effect differs with age from 1st through 12th grade of school [36]. Short-term memory was found to be involved in and to affect phonemic manipulations at all grade levels: the longer the manipulated stimulus, the poorer the performance. The finding is in line with the “transparency-by-modularity” (the concept “modularity” is referring to the flexibility of word order in the languages) interaction and suggests that Arabic is a “semi-modular” language in contrast to highly modular Hebrew. A theory to account for the acquisition of literary Arabic at an early age has been proposed based on the study results and previous findings. In a further study, Ibrahim et al. (2013) hypothesized that the superiority of balanced bilinguals would be greater in cognitive tasks which required spontaneous cognitive flexibility due to the fact that spontaneous cognitive flexibility required more cognitive resources and more advanced executive abilities than reactive cognitive flexibility [37]. In their study, Ibrahim et al. (2013) tested three groups of adults with a series of tests designed to tap the two types of cognitive flexibility: the experimental groups comprised bilinguals equally proficient in Hebrew and English (balanced), Hebrew-dominant bilinguals and English-dominant bilingual participants. The results showed that balanced bilinguals performed significantly better relative to individuals from the control group with the same cultural (mother tongue language) background. In both types of flexibility tasks, the balanced bilinguals were found to be superior to the Hebrew-dominant group but not compared to those who had mastered English as their primary language (English-dominant in the expression). A significant difference between the balanced-bilingual group and the Hebrew-dominant group was found in the task which required spontaneous cognitive flexibility and the one which required reactive cognitive flexibility. In this context, it has been proposed that EF mediates reading processes by facilitating the synchronization between auditory and visual abilities and the amount of attention allocated to the reading process [38]. Moreover, other studies conducted involving Hebrew-dominant children also showed significant positive correlations between reading proficiency in English-dominant children and Hebrew and EF skills [38,39]. Taken together, these findings strengthen the notion of EF involvement in the reading process in both Hebrew and English. However, Arabic, where the phonological representation of the written forms does not directly correspond to the spoken ones, is very unique and needs further investigation.

On the other hand, the literature has mixed results that put into question the existence of a bilingualism advantage in executive functioning [40,41,42,43].

In their study, De Bruin et al. (2014) argued that studies challenging the bilingual advantage were published the least, leading to a publication bias [40]. Recently, Lowe et al. (2021) contributed to the debate about whether bilingual children have an advantage in executive functioning relative to monolingual children [43]. According to their meta-analysis, they suggested that “the bilingual advantage in children’s executive functioning is small, variable, and potentially not attributable to the effect of language status”. In that regard, a former study of Abdelgafar and Moawad (2015) showed no significant differences between the groups across most executive function measures [44]. Also, in recent research, Alrwaita et al. (2022) assumed that cognitive effects of bilingualism beyond language had yielded inconsistent findings due to uncontrolled variables, such as linguistic similarities and the conversational settings in which bilinguals operate, including the opportunities they have to switch between languages [45]. In their study Alrwaita et al. (2022) focused on the cognitive effects of diglossia, where two varieties of the same language are spoken in distinct, well-defined contexts. Using linear mixed models, they compared the cognitive performance of Arabic-speaking diglossic young adults and English-speaking monolinguals on tasks assessing executive functions such as inhibition and switching. Although both groups performed as expected on all tasks, no significant effects of diglossia were found in these cognitive domains. They interpreted these results within the framework of the adaptive control hypothesis, suggesting that any influence on executive functions arising from the use of multiple languages or varieties may be less evident in situations where switching opportunities are limited, especially among younger adults. In further research, Alrwaita et al. (2024) tried to explore the impact of using two languages on executive functions by comparing the cognitive performance of three groups of older adults: Arab diglossic speakers, bilinguals, and monolinguals [46]. Participants were assessed using the Flanker and Stroop tasks, which measure inhibition skills, and the Color–Shape task, which evaluates switching abilities. The results revealed a cognitive advantage for diglossic speakers in the Flanker task, specifically in terms of inhibition, while no such benefit was found for the bilingual group. In a related issue, Tarabeh et al. (2023) explored how diglossia in Arabic affects phonological working memory among early readers [47]. Arabic-speaking first graders were examined in phonological working memory tasks, which were each administered in two language forms (spoken and standard Arabic). Two tasks focused on verbal retrieval, while one assessed the ability to remember instructions. The results revealed significant differences between the spoken and standard language stimuli in verbal retrieval tasks, but there were no notable differences in the task involving instruction recall, particularly as processing demands increased. The authors concluded that “the findings highlight the importance of developing more sensitive research tools and tasks to better assess the effects of diglossia on memory functions, particularly in relation to verbal working memory”.

### 3.2. Working Memory and Reading Arabic

The basic assumption that reading in Arabic differs from reading other languages came from studies that showed different cognitive load during reading in Arabic. Support for the involvement of EF during reading in Arabic came when subjects were presented with the words in the visual domain compared to the auditory domain [48,49]. Bentin and Ibrahim (1996) showed a greater effort and increased monitoring processes involved in the written form of Arabic. Diaz and Klingler (2000) reported better EF in bilinguals (visual–spatial skills, metalinguistic awareness, selective attention, and cognitive flexibility) compared to monolinguals. As working memory plays a central role in language (spoken and written) learning, if it is weak, learning can be considerably improved by selecting material that places less burden on the working memory. Numerous studies have found that good readers are more successful in STM tasks than weak readers, who find it difficult in the short term to retain verbal material presented either aurally or visually [42]. The reduced range of memory in the weak readers appears in a variety of verbal material such as numbers, letters, phrases, and sentences. The performance of these tasks depends on the efficient storage of information in a phonological code, which lessens the burden on the WM so that it can be freely occupied in the process of higher understanding. Thus, in addition to deficient phonological awareness, weak readers also have weaker short-term verbal memory, although it is still not clear which of the various processing components is responsible for this. Many authors attribute it to the ability to store unfamiliar phonological forms [50]. Tallal (1980) claimed that a functional lack of auditory temporal processing may cause a specific deficit in the organization of phonological information in the mind [51]. According to Tallal’s theory, even the difficulty in rapid manipulation—slowness in carrying out tasks of rapid manipulation, say retrieving the names of objects, colors, numbers and letters—is due to phonological deficiency, and does not indicate additional defects, as claimed by theories that attribute this to difficulties in automatization [52]. With repeated and extensive practice at processing information, some tasks require less effort and become more automatic. That is, when such skills become automatic, the brain is relieved of having to process individual units of information. This allows the brain to perform more complex processing and problem-solving tasks. It also improves the efficiency of the working memory system. The question that the researches tried to answer in this section is “to what extent do the characteristics of a language like Arabic, require different functioning of memory so that the long-term memory can function actively, while its contents support com—prehension during reading a text?”.

Several studies have made the connection between the efficiency of basic linguistic mechanisms (phonology, orthography) and the efficiency of cognitive processes of reading, such as memory and working memory (WM) [50,53]. Share and Stanovich (2000) compared typically developing and dyslexic children, and they concluded that “the dyslexics’ problems in deciphering and organizing phonological information are likely to cause difficulties in the storage and retrieval of words in short and long memory”. They also assumed that “difficulties may in turn impair the effectiveness with which the individual makes connections between the auditory and graphical components of written words, and the development of orthographic patterns. As a result, the processing of written words will be inefficient and impose a burden on the working memory”.

Earlier studies of reading acquisition of Arabic showed that it is slower than reading acquisition of Hebrew due to diglossia [1,2]. In a neurocognitive study using a lateralized matching technique, Eviatar et al. (2004) reported that “they found a pattern that supports the hypothesis that the left hemisphere (LH) is more sensitive to the local aspects of visual stimuli and the right hemisphere (RH) is more sensitive to the global aspects of these stimuli [54]. These results show that the RH cannot discriminate between letters that differ only in the placement or number of dots (e.g., /t/ت), while the LH can”. Following research focused on the contribution of graphemic complexity to the inhibition and slowness of reading acquisition, Taha and his colleagues (2013) reported that “such factors affect cognitive abilities, like memory components” [55].

In a subsequent study, Ibrahim (2011) reported that he “examined the influence of the diglossic nature of the Arabic language on the development of phonological ability, which is important for the proper development of reading” [36]. Phonological tasks were undertaken in both SA and LA to assess the WM skills measured at the end of each school year in 1st grade through 12th grade. Ibrahim (2011) hypothesized that “the participants would perform the tasks better with spoken words, to which they are more frequently exposed, than literary words; and that the influence of the location of the target phoneme will be more emphasized in the younger age groups”. The results revealed a clear advantage to executing spoken words over literary words. This finding is compatible with the findings of Saiegh-Haddad (2003) and might reflect stronger and more consolidated semantic connections in the spoken language and stronger control over the phonemes and phonological and syllabic structures belonging to the spoken language [3]. The phonemic deletion task, on the other hand, yielded a different trend. This dissociation is explained by the fact that the orthographical structures inherent in the phonemic analysis are more dependent on auditory modes [22]. Ibrahim (2011) reported that “performing better on literary language makes sense with this task as one can picture the words in text before segmenting and assembling”.

In a recent study, Maroun et al. (2020) examined typically reading and dyslexic adolescents undertaking phonological tasks in Arabic [56]. The unique characteristics of Arabic were hypothesized to affect dyslexic performance, such as the diglossic situation and the high visual complexity of Arabic orthography. The results revealed that as in other languages, phonological abilities are the best predictor for reading ability. Interestingly, it was found that the depth of the orthography had the same effect on typical and dyslexic readers.

## 4. Conclusions

The literature has shown that bilingualism has a positive effect on the functioning of an individual’s attentional system across the lifespan [31,32]. This review article highlights the contribution of diglossia as a form of bilingualism to the development of executive functions (EFs) by trying to examine whether the utilization of EF in Arabic-speaking children is domain specific (in reading) or domain general. Evidence from the literature supported the notion that the bilingual effect (of non-diglossic languages) seems to appear when assessing inhibition and cognitive flexibility but it disappears when working memory is considered [52]. However, in regard to Arabic, working memory has been proposed to mediate reading processes by facilitating the synchronization between auditory and visual abilities and the amount of attention allocated to the reading process [38]. Hence, the degree of involvement of linguistic skills in written language may differ because of the unique characteristics of the language examined. This is the case with Arabic, which exhibits unique diglossic features when the lexical distance between the two forms of Arabic has an adverse effect on the acquisition of basic reading skills [57,58,59] and other linguistic skills [60].

What are the cognitive requirements for a bilingual reader? And how is this related to the “adaptive control hypothesis”, which assumes that the simultaneous acquisition of several languages as a task is more cognitively demanding than the acquisition of a single language? [61]. This hypothesis, which received neurobiological support, argues that bilingualism demands the ability to manage linguistic interference, inhibition, monitoring and switching and that there is a reliance on cognitive control networks to achieve that [30].

### Implications and Limitations

The current review article provides insights into how Arab speaker–writers, who live in diglossic environments, benefit from the development of executive functions (EFs). The results manifested a variety of related differences. Although Arab children evince high levels of metalinguistic abilities, this advantage does not translate into an advantage in single-word reading acquisition. One central factor that could block the facilitating effect of phonological awareness is the visual complexity of Arabic orthography [55,62]. From a pedagogical point of view, it could have implications for teaching how to use the two language forms (categories) in the development of metacognitive skills in a diglossic environment. This may contribute to the modification of school curricula to expand this knowledge, resulting in enhancing learners’ abilities to improving MSA literacy skills. This means that education designers should improve curricula with the goal of prompting the use of spoken MSA in diverse situations, focusing on activities related to expressing opinions, conversations and different social meetings.

Given that the literature is relatively new in this area of research, the conclusions raised here might not seem warranted. This study examined the differences at the micro-level of linguistic production, investigating this issue at the level of single-word processing. Hence, future research is required to find out differences at the macro-structural level, with respect to the sentence and text level organization, narrative coherence, and discourse structure. In addition, any relevant future study should examine these issues so that it could give a more comprehensive illustration of the differences between the varieties and modalities of the two forms of Arabic.
